# Apigenin Prevents Ovarian Aging by Regulating Ca^2+^-Mediated Endoplasmic Reticulum Stress in Laying Chickens

**DOI:** 10.3390/antiox15030323

**Published:** 2026-03-04

**Authors:** Wanyue Gao, Jing Dong, Yingyu Xiao, Xiangyu Cai, Zhaoyu Yang, Weidong Zeng, Caiqiao Zhang, Yuling Mi

**Affiliations:** Department of Veterinary Medicine, College of Animal Sciences, Zhejiang University, Hangzhou 310058, China; 22317120@zju.edu.cn (W.G.); jingd@zju.edu.cn (J.D.); 22317125@zju.edu.cn (Y.X.); 22317105@zju.edu.cn (X.C.); 12117050@zju.edu.cn (Z.Y.); zengwd@zju.edu.cn (W.Z.)

**Keywords:** apigenin, Ca^2+^ homeostasis, ERS, ovarian aging, chickens

## Abstract

The sustainability of egg production in the poultry industry is frequently challenged by the progressive decline in ovarian function as laying chickens age. A primary driver of this reproductive transition is the functional deterioration of small white follicles (SWFs), which constitute the vital pre-hierarchical follicular reserve necessary for sustained egg production. However, the molecular mechanisms underlying age-associated SWF atresia remain poorly understood. In this study, we investigated the protective efficacy of apigenin (AP), a natural bioactive flavonoid, in mitigating follicular senescence by targeting calcium ion (Ca^2+^)-mediated endoplasmic reticulum stress (ERS) in D-galactose (D-gal)-induced SWFs and in naturally aged chickens. Our results revealed that AP treatment effectively rebalanced the D-gal-induced disruption of cell proliferation and survival. Molecular analysis of SWFs revealed that AP treatment promoted the coordinated restoration of transcriptional profiles of key Ca^2+^-handling genes, effectively counteracting the age-related disruption of ionic regulation. In addition, AP suppressed the aberrant upregulation of IP_3_R and modulated the expression of other key Ca^2+^-regulatory genes, including *CACNA1C*, *CACNA1D*, *CAMKII*, *MCU*, and *ATP2B1*. This restoration of intracellular Ca^2+^ homeostasis was associated with attenuation of the ERS response, as evidenced by the decreased levels of GRP78 and CHOP, and the suppression of Caspase-3-mediated apoptotic signaling. The biological relevance of these findings was further validated in vivo using naturally aged chickens. Dietary supplementation with AP significantly enhanced pre-hierarchical follicle recruitment in aged laying chickens, and improved egg production and eggshell quality in aged laying chickens. Collectively, these findings indicate that AP can modulate ERS signaling in laying chickens by maintaining intracellular Ca^2+^ homeostasis, thereby enhancing laying performance. These results highlight AP as a promising nutritional intervention to enhance reproductive performance and extend productive longevity in poultry.

## 1. Introduction

Reproductive longevity in commercial laying chickens is a primary determinant of economic efficiency in the poultry industry. The continuity of egg production is fundamentally governed by the hierarchical development of ovarian follicles, among which small white follicles (SWFs) serve as the primary follicular reserve for recruitment into the hierarchical follicle pool [1,2]. As hens enter the late laying period, a sharp decline in egg production is typically observed, driven primarily by reductions in both the quantity and quality of SWFs, together with an increased rate of follicular atresia. While follicular atresia is a programmed physiological process that regulates follicle number, an accelerated rate of atresia during aging contributes to premature depletion of the follicular pool [3]. Therefore, elucidating the molecular mechanisms that regulate follicular health in SWFs is vital for developing interventions to preserve ovarian function and extend the productive lifespan of poultry. Consequently, elucidating the molecular mechanisms underlying follicular health in SWFs is essential for developing strategies to maintain ovarian function and prolong the reproductive lifespan of poultry.

Current research indicates that ovarian aging is a complex process involving multiple pathways, including autophagy [4], ferroptosis [5], oxidative damage [6], and endoplasmic reticulum stress (ERS) [7]. Notably, excessive reactive oxygen species (ROS) can modify calcium ion (Ca^2+^)-handling proteins, leading to Ca^2+^ leakage from the ER and subsequent ERS [8]. The maintenance of a robust homeostasis network is essential for cellular integrity, yet its progressive decline is recognized as a fundamental hallmark of biological aging [9]. In the context of the ovary, oxidative stress disrupts the delicate balance of oxidative protein folding, leading to a loss of proteostasis that often manifests as chronic ERS, which subsequently compromises follicular function and accelerates the depletion of the ovarian reserve [10]. ERS occurs when the protein-folding capacity of the endoplasmic reticulum (ER) is overwhelmed, thereby activating the unfolded protein response (UPR) to reestablish cellular homeostasis [11]. Although ERS has been reported in mammalian models and aged laying chickens, the crosstalk between Ca^2+^ homeostasis, ERS, and their combined influence on ovarian and follicular function in laying chickens remains largely unexplored [7,12]. Recent evidence suggests that Ca^2+^ signaling plays a critical regulatory role in this stress response [13,14]. Intracellular Ca^2+^ levels are dynamically maintained through channels such as the IP_3_R, yet the link between Ca^2+^ dysregulation and ERS during follicular aging in chickens remains to be fully elucidated.

To mitigate the decline in reproductive performance during the late laying period, increasing attention has been directed toward dietary supplementation with natural plant extracts to support ovarian function. Apigenin (AP), a dietary flavonoid found in high concentrations in parsley and celery, has been identified as a promising candidate for nutritional interventions aimed at improving reproductive function. This compound exhibits potent antioxidant activity by enhancing cellular antioxidant defense systems [15]. By scavenging free radicals and upregulating endogenous antioxidant enzymes, AP helps maintain the redox equilibrium necessary for oocyte and granulosa cell (GC) survival [16]. In poultry models, AP supplementation has been reported to mitigate oxidative stress, enhance antioxidant enzyme activity, and improve ovarian function, in part by preserving cellular membrane integrity [17]. In broilers with necrotic enteritis (NE), dietary AP mitigated growth retardation, restored intestinal barrier integrity, and modulated immune and antioxidant responses, thereby alleviating NE-induced intestinal damage and systemic stress [18]. Moreover, in dairy cattle, AP has been shown to modulate GPCR signaling, thereby promoting ovarian follicle development, luteinisation, and overall reproductive performance [19]. Despite these known benefits, whether AP can maintain Ca^2+^ homeostasis and alleviate ERS in the aging avian ovary remains to be determined.

Given AP’s potential role in maintaining Ca^2+^ balance and alleviating cellular stress, we hypothesized that AP exerts protective effects against follicular senescence in both naturally aged chickens and D-galactose (D-gal)-induced senescence model in SWFs. We investigated the effects of AP on Ca^2+^ homeostasis and ERS signaling in SWFs and in the ovaries of laying chickens. This study aims to elucidate the cellular mechanisms by which AP preserves ovarian function through regulation of Ca^2+^ homeostasis and ERS signaling, thereby contributing to improved reproductive performance during the late laying period.

## 2. Materials and Methods

### 2.1. Animals and Sample Collection

Hy-Line White laying chickens were sourced from Huajie Poultry Company (Hangzhou, China). For the in vivo experiment, 192 Hy-Line White chickens, aged 480 days, were used and randomly allocated to four dietary treatments. Chickens in the control group were offered the basal diet, whereas the other three treatments received the same diet containing different doses of AP (100, 300, and 500 mg/kg, 520-36-5, Jinrun Biotech, Taiyuan, China). Each treatment group comprised 48 chickens housed in 6 pens, with 8 birds per pen to ensure uniform stocking density. The trial was conducted under controlled environmental conditions at an ambient temperature of 28–32 °C. Throughout the experimental period, birds had ad libitum access to feed and drinking water. Chickens were acclimatized to the environment for 7 days prior to experiments.

Based on evidence that AP supplementation at 500 mg/kg alleviates oxidative stress in chickens [20] and does not cause toxicity at higher doses [21]. Therefore, considering the effective dosage range of other flavonoids in used poultry [22], AP was supplemented at 100, 300, and 500 mg/kg.

At the conclusion of the trial, five chickens from each replicate were randomly selected, and their body weights were recorded. Subsequently, chickens were euthanized by exsanguination from the jugular vein after anesthesia induced by intravenous injection of sodium pentobarbital (40 mg/kg BW). The intact ovary was excised, and individual follicles were isolated and classified [23]. Specifically, follicles were categorized by diameter into: small white follicles (SWFs, <2 mm), large white follicles (LWFs, 2–4 mm), and small yellow follicles (SYFs, 4–8 mm), alongside the hierarchy of preovulatory follicles (Pre, >10 mm). Ovarian cortex, SWFs, and serum were collected and stored in cryostorage tubes and submerged in liquid nitrogen for preservation, while egg production was monitored daily throughout the experimental period.

### 2.2. Tissue Culture

For the in vitro experiments, SWFs (diameter < 2 mm) were collected from ovaries of 280-day-old (D280) peak-laying chickens. Chickens were euthanized by exsanguination from the jugular vein after anesthesia induced by intravenous injection of sodium pentobarbital (40 mg/kg BW). Whole follicles were cultured in 24-well plates containing high-glucose DMEM (HyClone, Tauranga, New Zealand) supplemented with 5% fetal bovine serum (HyClone, Tauranga, New Zealand) and 1% penicillin-streptomycin (HyClone, Tauranga, New Zealand). The follicles were maintained at 38.5 °C in a humidified atmosphere with 5% CO_2_, following the procedure previously described [24].

### 2.3. Drug Treatment

D-gal (MB1853, MeilunBio, Dalian, China) was prepared by dissolving the powder directly in high-glucose DMEM. AP (purity ≥ 98%, CAS520-36-5, Yuanye, Shanghai, China) was first solubilized in dimethyl sulfoxide (DMSO) and then diluted with culture medium so that the final DMSO content did not exceed 0.1%. SWFs were pretreated with a 10 µM concentration of AP for 24 h. A membrane-permeant myo-inositol-1,4,5-trisphosphate derivative (10 µM IP_3_/AM, CAT3-1-145, SiChem GmbH, Bremen, Germany) was employed as an IP_3_R agonist for the inositol 1,4,5-trisphosphate receptor (IP_3_R), a critical calcium release channel on the endoplasmic reticulum. Cellular senescence was then established by incubating SWFs with 200 mM D-gal for 48 h, with the medium being refreshed every 24 h.

### 2.4. RNA Sequencing

SWF samples for in vitro were collected after culture, snap-frozen in liquid nitrogen, and submitted to Novogene (Beijing, China) for RNA sequencing. Total RNA was extracted, poly(A) mRNA was enriched, libraries were constructed and quality-checked, and samples were sequenced on an Illumina platform. Raw reads were adapter-trimmed and quality-filtered and aligned to the Gallus gallus reference genome (version per vendor report) using spliced transcripts alignment to a reference (STAR). Gene-level counts were obtained with featureCounts v1.5.0-p3. Differential expression was performed in R using differential expression sequencing 2 (DESeq2) v1.20.0 after filtering low-abundance genes. Significance was set at false discovery rate (FDR) < 0.05 and |log_2_ Fold Change| ≥ 1. Variance-stabilized expression values were used for data visualization, including heatmaps with hierarchical clustering and volcano plots. Functional enrichment analysis of differentially expressed genes (DEGs) was conducted using clusterProfiler v3.8.1 with Kyoto Encyclopedia of Genes and Genomes (KEGG) pathways annotations, considering terms/pathways significant at FDR < 0.05.

### 2.5. Hematoxylin and Eosin Staining

After fixation in 4% paraformaldehyde (BL539A, Biosharp, Shanghai, China) for 24 h, SWFs and ovarian cortex were rinsed under running water overnight, passed through a graded ethanol series for dehydration, and then infiltrated with paraffin for 40 min before embedding. Paraffin blocks were sliced into 5 μm sections, which were stained with hematoxylin and eosin (H&E) according to standard histological procedures. The stained sections were examined and imaged using an Eclipse 80i light microscope (Nikon, Tokyo, Japan).

### 2.6. TUNEL Staining

TdT-mediated dUTP Nick-End Labeling (TUNEL)-positive apoptotic cells in SWFs were identified in ovarian cortex and SWF paraffin sections (5 μm thickness) using a TUNEL apoptosis assay kit (A112-03, Vazyme, Nanjing, China) according to the manufacturer’s instructions. Three SWFs or ovaries were randomly selected from each of the three independent biological replicates per group. Following counterstaining with 4′,6-diamidino-2-phenylindole (DAPI) (MA0128, MeilunBio) to visualize cell nuclei, fluorescent images were captured using an IX70 fluorescence microscope (Olympus, Tokyo, Japan).

Quantitative analysis of apoptosis was performed using ImageJ v1.44. Three random fields per section (558 × 419 μm) were acquired at a resolution of 1920 × 1440 pixels. For each field, TUNEL-positive cells were isolated by applying a uniform threshold. The apoptosis level was then determined by measuring the Mean Fluorescence Intensity within these thresholded regions. Results are expressed as fold changes relative to the control group, which was normalized to 1.

### 2.7. BrdU Staining

The tissue sections were washed with phosphate-buffered saline (PBS), fixed with 10% methanol for 5–10 min and then treated with 0.1 M hydrochloric acid for DNA denaturation, followed by washing with PBS. The sections were then incubated with a 10 μM 5-bromo-2′-deoxyuridine (BrdU) labeling solution at room temperature for 1–2 h, followed by a PBS wash. Next, the sections were incubated with an anti-BrdU antibody (HA601120, HuaBio, Hangzhou, China) for 1 h at room temperature or overnight at 4 °C, then incubated with appropriate secondary antibodies and counterstained with DAPI. Fluorescent images were captured using an IX70 fluorescence microscope. ImageJ v1.44 software was used to quantify the BrdU-positive cell rate in SWFs.

Quantitative analysis of cell proliferation was performed using ImageJ v1.44. Three random fields per section (558 × 419 μm) were acquired at a resolution of 1920 × 1440 pixels. For each field, BrdU-positive cells were isolated by applying a uniform threshold. The proliferation level was then determined by measuring the Mean Fluorescence Intensity within these thresholded regions. Results are expressed as fold changes relative to the control group, which was normalized to 1.

### 2.8. Transmission Electron Microscope Assay

For ultrastructural analysis, cultured SWFs were collected and fixed overnight at 4 °C with 2.5% glutaraldehyde in 0.1 M phosphate buffer (pH 7.4). Specimens were dehydrated through a graded ethanol series (30–100%), followed by pure acetone, then embedded in epoxy resin and polymerized at 60 °C for 48 h. Ultrathin sections (approximately 70 nm) were prepared using an ultramicrotome (Leica EM UC7, Wetzlar, Germany), stained with uranyl acetate followed by Reynolds’ lead citrate, and examined on a Tecnai G2 Spirit transmission electron microscope (TEM; FEI, Hillsboro, OR, USA) to document the ultrastructure of ER and mitochondria in GCs of SWFs.

### 2.9. RNA Extraction and RT-qPCR

SWFs were rinsed with pre-cooled PBS and then homogenized using TRIzol-based reagent (TaKaRa, Dalian, China). Afterward, total RNA was isolated, and its concentration and purity were determined using a NanoDrop 2000 spectrophotometer (Thermo Fisher Scientific, Waltham, MA, USA) based on the A260/A280 ratio. Subsequently, 1 µg of total RNA was reverse-transcribed into cDNA using the HiScript II 1st Strand cDNA Synthesis Kit (Vazyme, Nanjing, China). Real-time quantitative PCR (RT-qPCR) was performed using the ChamQ Universal SYBR qPCR Master Mix (Vazyme, Nanjing, China) with a reaction system consisting of cDNA template, specific primers, and SYBR Green mix in 96-well plates. The thermal cycling conditions were: 95 °C for 5 min, followed by 40 cycles of 95 °C for 15 s and 60 °C for 1 min, followed by a melting curve analysis to verify amplification specificity [5]. The relative expression levels of target genes were calculated using the 2^−ΔΔCt^ method, with β-actin used as the internal reference gene. Primer sequences are listed in Table S1.

### 2.10. Western Blot Analysis

SWFs were homogenized with Radio Immunoprecipitation Assay (RIPA) Buffer (Fdbio, Hangzhou, China) supplemented with 1% protease inhibitor (A1320914, AmBeed, Shanghai, China) and phosphatase inhibitor (A2679231, AmBeed, Shanghai, China) cocktails. After centrifugation, the supernatant was collected. Protein separation and immunoblotting were performed as previously described [24]. To ensure the specificity of the immunoreaction, all primary antibodies used were commercially validated by the manufacturer for Western Blot application. The primary antibodies used in this study included rabbit anti-PCNA (ET1605-38, Huabio, Hangzhou, China), rabbit anti-BAX (ER0907, Huabio, Hangzhou, China), rabbit anti-IP_3_R (ET1704-77, Huabio, Hangzhou, China), rabbit anti-GRP78 (ER1706-50, Huabio, Hangzhou, China), rabbit anti-CHOP (ET1703-05, Huabio, Hangzhou, China), rabbit anti-CACNA1C (ER1803-49, Huabio, Hangzhou, China), rabbit anti-β-Tubulin (ET1609-48, Huabio, Hangzhou, China). The specificity was further confirmed by the detection of single bands at the predicted molecular weights. Additionally, negative controls omitting the primary antibodies were processed simultaneously to rule out non-specific binding of the secondary antibody. The chemiluminescent signal was captured, and band density was quantified with ImageJ. The expression of all proteins was normalized to β-Tubulin.

### 2.11. Measurement of Intracellular Ca^2+^ Concentration

Intracellular Ca^2+^ levels were determined using a Fluo-4 Calcium Ion Detection Kit (S1061S, Beyotime, Shanghai, China) following the manufacturer’s instructions. After culture, SWFs were collected and washed twice with phosphate-buffered saline (PBS). The SWFs were then dissected, and the GC layer was carefully separated using surgical forceps. The isolated granulosa layer was rinsed with PBS to remove residual yolk, followed by enzymatic digestion in 1 mg/mL collagenase at 37 °C for 3 min with gentle pipetting. The resulting suspension was filtered through a 200-mesh cell strainer to obtain a single-cell suspension. The digestion was terminated by adding an equal volume of complete medium. The cells were centrifuged at 8000 rpm for 5 min at room temperature, and the supernatant was discarded. The cell pellet was washed once with PBS and centrifuged again under the same conditions. The cell pellet was resuspended in 200 µL of Fluo-4 working solution to prepare a single-cell suspension. Cells were incubated at 37 °C for 30 min in the dark. After incubation, fluorescence was recorded using a flow cytometer.

### 2.12. Statistical Analysis

All experiments were performed with at least three independent biological replicates. Data are expressed as mean ± standard error of the mean (SEM), as indicated in figure legends. Statistical analysis was conducted using one-way analysis of variance (ANOVA) with Tukey’s multiple comparisons test or Student’s *t*-test using the SPSS 20.0 software, and data visualization was performed with GraphPad Prism 10.1.2. A value of *p* < 0.05 was considered statistically significant.

## 3. Results

### 3.1. AP Alleviates D-Gal-Induced Apoptosis and Promotes Cell Proliferation in SWFs

**Figure 1:**
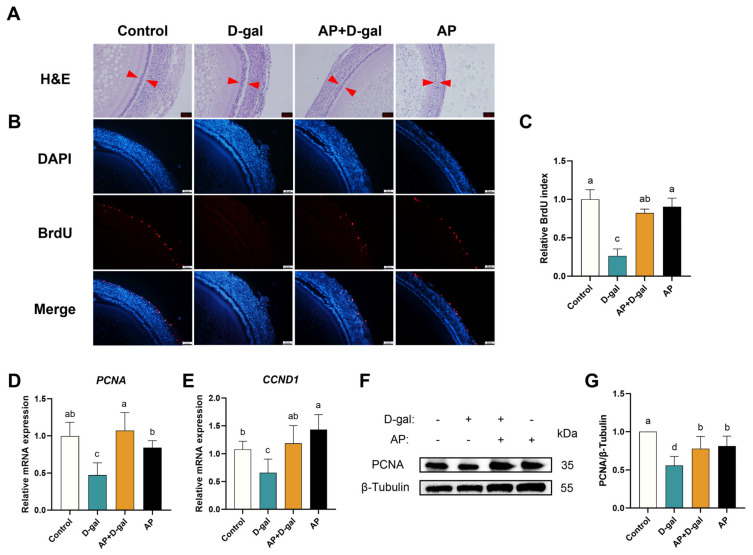
Effect of AP treatment on follicular morphology and cell proliferation in D-gal-induced SWFs. (**A**) H&E staining of SWFs. Scale bar: 50 μm. Red arrow: GC layer. (**B**) BrdU staining of SWFs. BrdU-positive cells in red, nuclei in blue (DAPI). Scale bar: 50 µm. (**C**) Relative BrdU index. (**D**,**E**) RT-qPCR analysis of *PCNA* and *CCND1* mRNA expression levels. (**F**,**G**) Western blot detection and analysis of protein expression level (*PCNA*). Data are presented as mean ± SEM (*n* ≥ 3). Protein and mRNA expression levels were normalized to the control group. Significant differences between groups are indicated by different lowercase letters (*p* < 0.05).

Using the D-gal-induced SWF aging model, we performed histological and molecular analyses to evaluate the protective effects of AP on senescent follicles. H&E staining revealed that follicles in the D-gal group exhibited marked disruption of GC layer integrity, characterized by disorganized and loosely arranged cells. In contrast, AP treatment preserved normal follicular architecture and re-established a compact GC layer structure (Figure 1A). Cellular proliferation, assessed by BrdU staining, showed a significant reduction in the BrdU-positive cell index in the D-gal group compared with the control, whereas AP treatment restored the BrdU-positive cell index to near-control levels (Figure 1B,C). Consistent with these observations, the mRNA expression levels of proliferation-associated genes PCNA and CCND1 were markedly downregulated following D-gal exposure, but were effectively rescued by AP treatment (Figure 1D,E). Notably, CCND1 expression in the AP-only group exceeded that of the control group, suggesting a potential stimulatory effect of AP on follicular proliferation. Western blot analysis further demonstrated that D-gal significantly suppressed PCNA protein expression relative to the control group, while AP treatment counteracted this inhibition and restored PCNA levels to baseline (Figure 1F,G).

**Figure 2:**
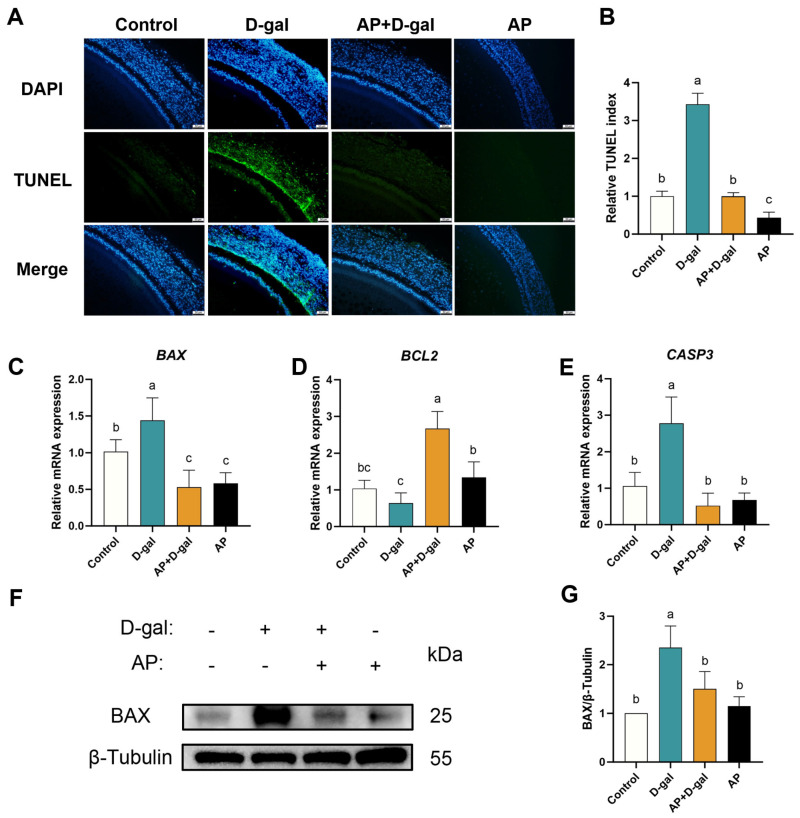
Effect of AP treatment on apoptosis in D-gal-induced SWFs. (**A**) TUNEL staining of SWFs. Apoptotic cells in green (TUNEL), nuclei in blue (DAPI). Scale bar: 50 µm. (**B**) Relative TUNEL index. (**C**–**E**) RT-qPCR analysis of *BAX*, *BCL2*, and *CASP3* mRNA expression levels. (**F**,**G**) Western blot detection and analysis of protein expression level (*BAX*). Data are presented as mean ± SEM (*n* ≥ 3). Protein and mRNA expression levels were normalized to the control group. Significant differences between groups are indicated by different lowercase letters (*p* < 0.05).

The effects of AP treatment on apoptosis in D-gal-induced SWFs are shown in Figure 2. TUNEL staining revealed a marked increase in apoptotic cells in the D-gal group compared with the control, whereas AP treatment significantly reduced the proportion of TUNEL-positive cells relative to total nuclei (Figure 2A,B). The mRNA expression levels of apoptosis-related genes were further examined. AP treatment significantly downregulated the pro-apoptotic genes *BAX* and *CASP3*, and concomitantly upregulating the anti-apoptotic gene *BCL2* (Figure 2C–E). Consistent with the mRNA results, Western blot analysis further demonstrated that D-gal exposure significantly elevated BAX protein expression, whereas AP treatment effectively suppressed this increase (Figure 2F,G).

### 3.2. Transcriptomic Profiling Identifies Key Pathways Modulated by Apigenin in Senescent SWFs

**Figure 3:**
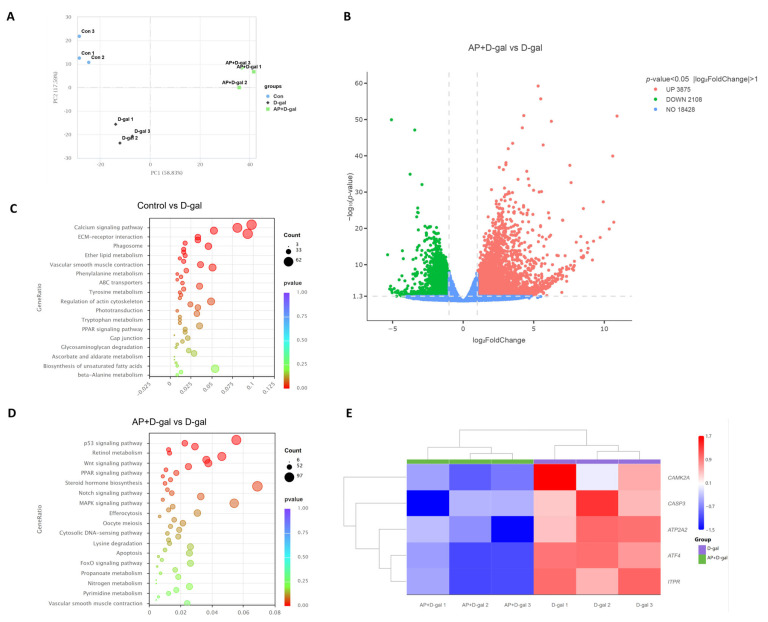
Transcriptomic Profiling of SWFs in vitro. (**A**) Principal component analysis of the control, D-gal, and AP+D-gal groups. (**B**) Volcano plot of DEGs between the AP+D-gal and D-gal groups. Downregulated genes are shown in green, upregulated genes in red, and non-significant genes in blue. The horizontal dashed line indicates the threshold for statistical significance (*p* < 0.05, corresponding to −log_10_(*p*-value) > 1.3). The two vertical dashed lines represent the thresholds for fold change (|log_2_FoldChange| > 1). (**C**) KEGG pathway enrichment analysis showing the top 17 significantly enriched KEGG pathways in the controls versus D-gal group. (**D**) KEGG pathway enrichment analysis showing the top 17 significantly enriched KEGG pathways in the AP+D-gal group versus D-gal group. Dot size represents the number of genes annotated to each pathway, and a color gradient from red to purple indicates the level of enrichment significance. (**E**) Hierarchical clustering heatmap of differentially expressed genes, with color intensity variation indicating relative expression levels.

To elucidate the molecular mechanisms underlying the protective effects of AP against D-gal-induced follicular damage, transcriptome sequencing was conducted on SWFs from the control, D-gal, and AP+D-gal groups. Principal component analysis (PCA) demonstrated a clear separation among the three groups along principal components 1 (PC1) and principal components 2 (PC2), indicating distinct transcriptional profiles (Figure 3A). Differential expression analysis between the AP+D-gal and D-gal groups identified 5983 DEGs, including 3875 upregulated and 2108 downregulated genes, while 18,428 genes were not significantly altered (Figure 3B). KEGG pathway enrichment analysis revealed distinct signaling alterations among groups. The D-gal group showed significant enrichment of the Ca^2+^ signaling pathway relative to the control (Figure 3C), while comparison between the AP+D-gal and D-gal groups indicated pronounced enrichment of ERS-associated apoptosis-related signaling pathways (Figure 3D). Hierarchical clustering analysis further revealed that genes involved in Ca^2+^ signaling and ERS pathways—such as *ITPR*, *ATF4*, *ATP2A2*, *CASP3*, and *CAMKII*—were markedly downregulated in the AP+D-gal group compared with the D-gal group (Figure 3E), suggesting that AP mitigates D-gal-induced follicular injury primarily through modulation of Ca^2+^ homeostasis and ERS-associated apoptotic signaling.

### 3.3. AP Treatment Maintain Ca^2+^ Homeostasis and Alleviates ERS in D-Gal-Induced SWFs

**Figure 4:**
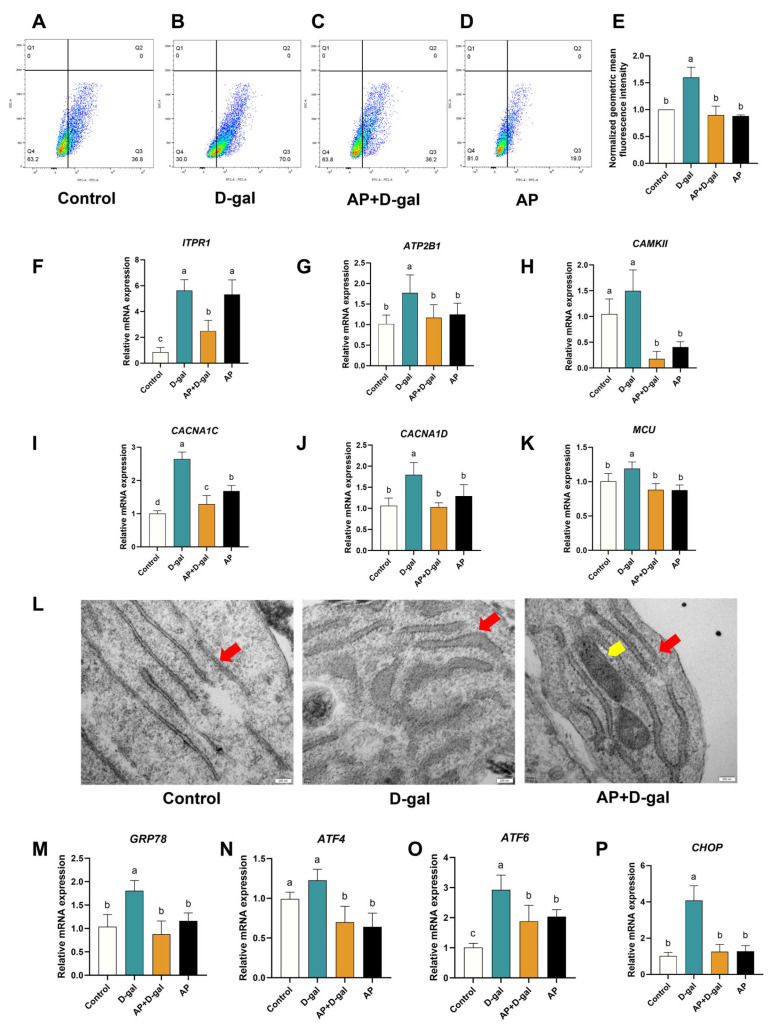
Effects of AP on Ca^2+^ homeostasis and ERS in D-gal-induced SWFs. (**A**–**D**) Flow cytometric analysis of intracellular Ca^2+^ levels. The pseudo-color density plot indicates the distribution of cell populations, where the color gradient from blue to red represents increasing cell density. The black vertical and horizontal lines indicate the **quadrant gates** used for population analysis. The numbers in each quadrant represent the percentage of cells within that specific gate. (**E**) Normalized geometric mean fluorescence intensity of Fluo-4 AM fluorescence. (**F**–**K**) RT-qPCR detection of mRNA expression levels (*ITPR1*, *ATP2B1*, *CAMKII*, *CACAN1C*, *CACNA1D*, and *MCU*). (**L**) TEM analysis of ER morphology in SWFs. Red arrows: ER. Yellow arrow: mitochondria. Scale bar: 200 nm. (**M**–**P**) RT-qPCR detection of mRNA expression levels of ER stress-related genes (*GRP78*, *ATF4*, *ATF6*, and *CHOP*). Data are presented as mean ± SEM (*n* ≥ 3). The mRNA expression levels are normalized to the control group. Significant differences between groups are shown by distinct lowercase letters in a test (*p* < 0.05).

To investigate whether AP treatment mitigates D-gal-induced disruption of Ca^2+^ homeostasis, GCs isolated from cultured SWFs were analyzed by flow cytometry. D-gal exposure significantly increased intracellular Ca^2+^ levels, as evidenced by an increased proportion of cells exhibiting high Fluo-4 AM fluorescence intensity (Figure 4A–D). AP treatment markedly attenuated this alteration, significantly reducing the proportion of cells with elevated intracellular Ca^2+^ levels. Consistent with these findings, quantitative analysis of normalized geometric mean fluorescence intensity showed a significant elevation in the D-gal group compared with the control group. In contrast, no significant differences were observed among the control, AP+D-gal, and AP groups (Figure 4E).

At the transcriptional level, D-gal treatment significantly upregulated multiple Ca^2+^ channel-related genes, including *ITPR1*, *ATP2B1*, *CAMKII*, *CACNA1C*, *CACNA1D*, and *MCU*, relative to the control. AP treatment effectively normalized the expression of these genes, with transcript levels in the AP+D-gal group restored to near-control levels. Notably, *ITPR1* expression remained elevated in the AP group and was not markedly reduced relative to the D-gal group (Figure 4F–K).

Ultrastructural examination of GCs by transmission electron microscopy revealed pronounced ER swelling following D-gal exposure, whereas AP treatment markedly alleviated this ER dilation (Figure 4L). Consistent with these morphological changes, the mRNA expression levels of ERS-related genes (*GRP78*, *ATF4*, *ATF6*, and *CHOP*) were significantly elevated in the D-gal group but were restored to near-control levels with AP treatment (Figure 4M–P).

### 3.4. IP_3_R Activation Negates the Protective Effects of Apigenin on Ca^2+^ Balance, ERS, and Apoptosis

**Figure 5:**
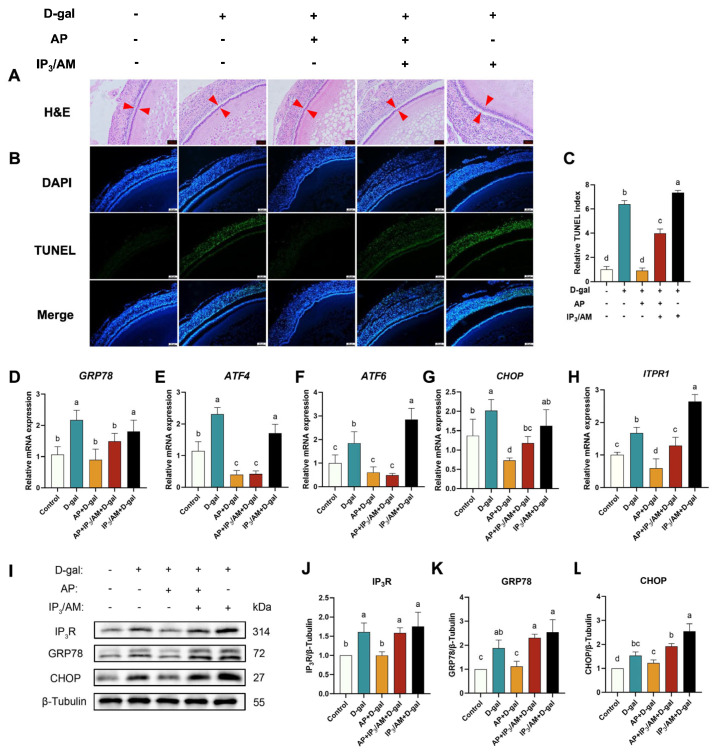
Effects of AP and IP_3_/AM on follicular morphology, apoptosis, and ERS in D-gal-induced SWFs. (**A**) H&E staining on SWFs. Red arrow: GC layer. Scale bar: 50 μm. (**B**) TUNEL staining of SWFs. Apoptotic cells in green (TUNEL), nuclei in blue (DAPI). Scale bar: 50 μm. (**C**) Relative TUNEL index. (**D**–**H**) RT-qPCR detection of mRNA expression levels (*GRP78*, *ATF4*, *ATF6*, *CHOP*, and *ITPR1*). (**I**–**L**) Western blot detection and analysis of protein expression levels (IP_3_R, GRP78, and CHOP). Data are presented as mean ± SEM (*n* ≥ 3). Protein and mRNA expression levels are normalized to the control group. Significant differences between groups are indicated by distinct lowercase letters (*p* < 0.05).

The protective effect of AP against D-gal-induced follicular aging was closely associated with modulation of the IP_3_R-mediated Ca^2+^ signaling pathway. To verify whether pharmacological activation of IP_3_R could negate the beneficial effects of AP on D-gal-induced apoptosis in SWFs, histological and apoptotic analyses were conducted. As shown in Figure 5A, D-gal treatment disrupted the structural integrity of GC layer, whereas AP treatment preserved normal follicular morphology. However, this protective effect was abolished following administration of IP_3_/AM.

TUNEL staining revealed that D-gal exposure markedly increased the proportion of TUNEL-positive apoptotic cells, while AP treatment significantly reduced this signal; notably, this reduction was reversed following IP_3_R activation (Figure 5B). Quantitative analysis further confirmed that the TUNEL index was significantly elevated in the D-gal group compared with the control, reduced by AP treatment, and subsequently increased following IP_3_/AM administration (Figure 5C).

Consistent with these morphological and apoptotic observations, mRNA expression levels of *GRP78*, *CHOP*, *ATF4*, *ATF6*, and *ITPR1* were markedly upregulated in the D-gal group. AP treatment significantly suppressed these D-gal-induced elevations, whereas IP_3_R activation reversed the inhibitory effects of AP (Figure 5D–H). Western blot analysis further demonstrated that D-gal exposure increased the protein abundance of IP_3_R, GRP78, and CHOP, all of which were substantially reduced by AP treatment. Moreover, these AP-mediated effects were abolished by IP_3_/AM (Figure 5I–L).

### 3.5. AP Supplementation Maintains Ca^2+^ Homeostasis and Alleviates ERS in SWFs of Aged Chickens

**Figure 6:**
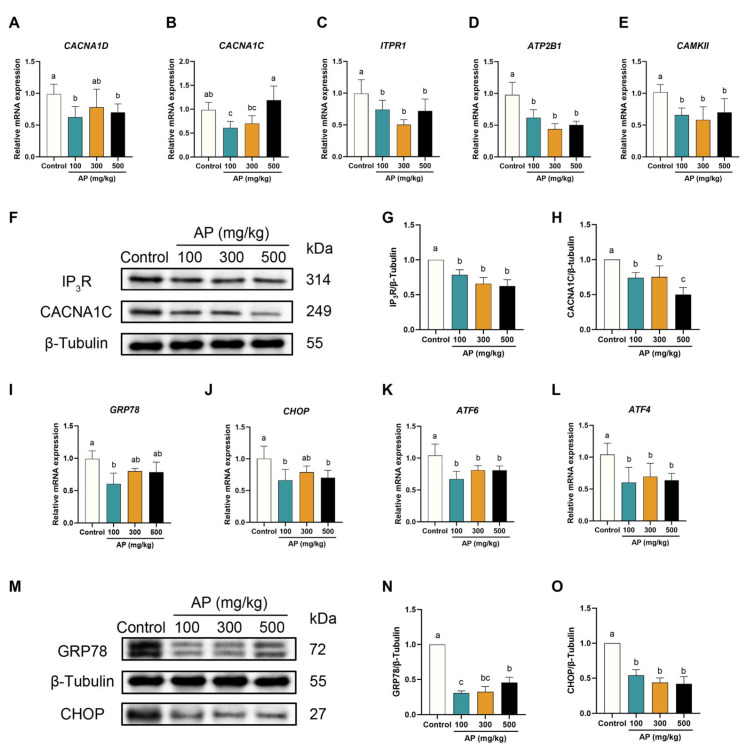
Effect of AP supplementation on the Ca^2+^ channel and ERS in SWFs of D480 chickens. (**A**–**E**) RT-qPCR detection of mRNA expression levels (*CACNA1C*, *CACNA1D*, *ITPR1*, *ATP2B1*, and *CAMKII*). (**F**–**H**) Western blot detection and analysis of protein expression levels (IP_3_R, CACNA1C). (**I**–**L**) RT-qPCR detection of mRNA expression levels (*GRP78*, *CHOP*, *ATF6*, and *ATF4*). (**M**–**O**) Western blot detection and analysis of protein expression levels (GRP78, CHOP). Data are presented as mean ± SEM (*n* ≥ 3). Protein and mRNA expression levels are normalized to the control group. Significant differences between groups are indicated by distinct lowercase letters (*p* < 0.05).

Compared with the control group, low-laying chickens receiving AP supplementation exhibited a significant downregulation of Ca^2+^ homeostasis-related genes. However, no significant changes were observed in *CACNA1D* or *CACNA1C* expression in the 300 mg/kg group, nor in *CACNA1C* expression in the 500 mg/kg group (Figure 6A–E). Consistently, the protein levels of the Ca^2+^ signaling regulators IP_3_R and CACNA1C were markedly reduced in AP-supplemented groups (Figure 6F–H). In parallel, AP supplementation effectively attenuated ERS in SWFs. The transcript levels of *GRP78*, *CHOP*, *ATF4*, and *ATF6* were significantly decreased relative to the control group; however, *GRP78* and *CHOP* expression in the 300 mg/kg group, as well as *GRP78* expression in the 500 mg/kg group, did not differ significantly from control levels (Figure 6I–L). At the protein level, both GRP78 and CHOP were notably downregulated in AP-supplemented groups, further confirming the alleviating effect of AP on ERS (Figure 6M–O).

### 3.6. AP Alleviates Apoptosis in SWFs and Ovary of Aged Chickens

**Figure 7:**
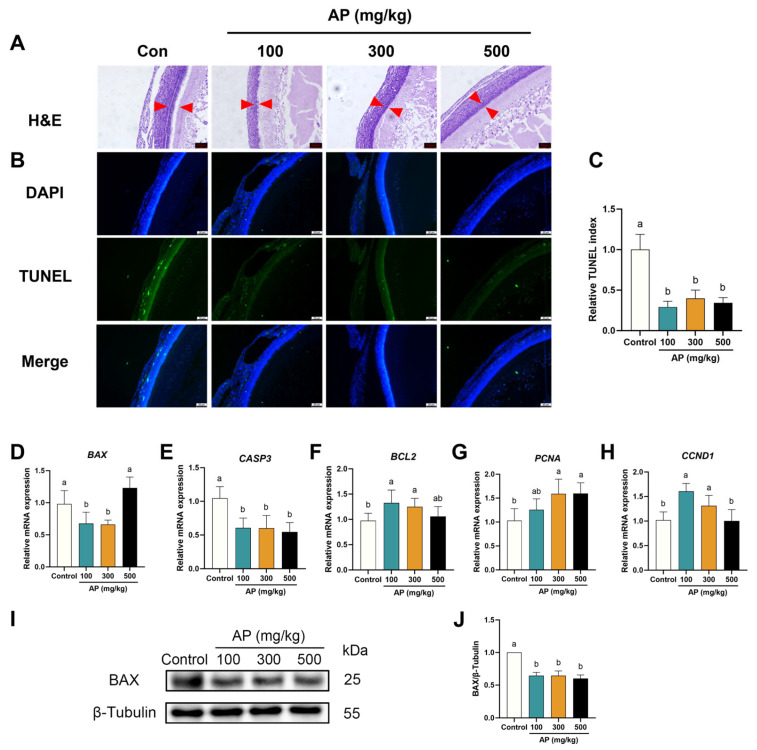
Effect of AP supplementation on follicular morphology and anti-apoptosis capacity in SWFs of D480 chickens. (**A**) H&E staining of SWFs. Scale bar: 50 μm. Red arrow: GC layer. (**B**) TUNEL staining of SWFs. Apoptotic cells in green (TUNEL), nuclei in blue (DAPI). Scale bar: 50 µm. (**C**) Relative TUNEL index. (**D**–**H**) RT-qPCR detection of mRNA expression levels (*BAX*, *CASP3*, *BCL2*, *PCNA*, and *CCND1*). (**I**,**J**) Western blot detection and analysis of protein expression levels (BAX). Data are presented as mean ± SEM (*n* ≥ 3). Protein and mRNA expression levels are normalized to the control group. Significant differences between groups are indicated by distinct lowercase letters (*p* < 0.05).

The effects of AP on SWFs morphology in low-laying chickens are shown in Figure 7. H&E staining indicated that, compared with the control group, GC layer cells in the AP-supplemented groups were more densely arranged and exhibited well-preserved, regular morphology (Figure 7A). TUNEL staining further demonstrated a significant reduction in the number of apoptotic cells in the AP-supplemented groups relative to the control (Figure 7B,C). The mRNA expression levels of apoptosis genes were further assessed. AP supplementation significantly downregulated the expression levels of *BAX* and *CASP3*, while upregulating the expression levels of anti-apoptosis related genes *BCL*2 in SWFs (Figure 7D–F). The expression levels of proliferation- and cell cycle-related genes *PCNA* and *CCND1* showed an increasing trend following AP supplementation compared with the control group (Figure 7G,H). Consistent with these findings, Western blot analysis revealed that the protein abundance of BAX was notably reduced following AP treatment (Figure 7I,J).

**Figure 8:**
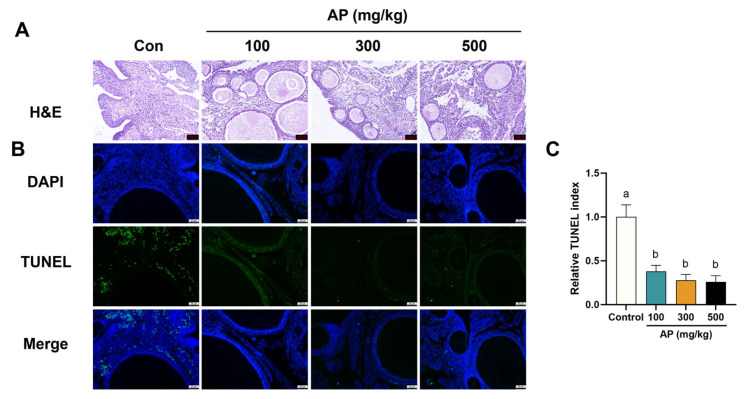
Effects of AP supplementation on ovarian morphology and apoptosis in D480 chickens. (**A**) H&E staining of ovarian tissues. Scale bar: 50 µm. (**B**) TUNEL staining of ovarian tissues. Apoptotic cells in green (TUNEL), nuclei in blue (DAPI). Scale bar: 50 μm. (**C**) Relative TUNEL index. Significant differences between groups are indicated by distinct lowercase letters (*p* < 0.05).

Similar morphological changes were observed in the ovaries. H&E staining demonstrated that AP supplementation increased the number of small follicles in the ovarian tissue of laying chickens compared with the control group. In parallel, the number of TUNEL cells in ovarian tissue was significantly lower in the AP-supplemented group than in the control group (Figure 8).

### 3.7. Effects of AP Supplementation on Serum Biochemical Parameters

The effects of dietary AP supplementation on serum biochemical parameters in low-laying chickens are presented in Table 1. No significant differences were detected among groups in the levels of alanine aminotransferase (ALT), aspartate aminotransferase (AST), γ-glutamyltransferase (GGT), albumin (Alb), globulin (Glob), or total protein (TP). In contrast, progesterone (P) concentrations exhibited a dose-dependent increasing trend in response to AP supplementation, with significant elevations observed in the 300 mg/kg and 500 mg/kg groups compared with the control group.

### 3.8. Effects of AP Supplementation on Egg Production and Follicle Development

**Figure 9:**
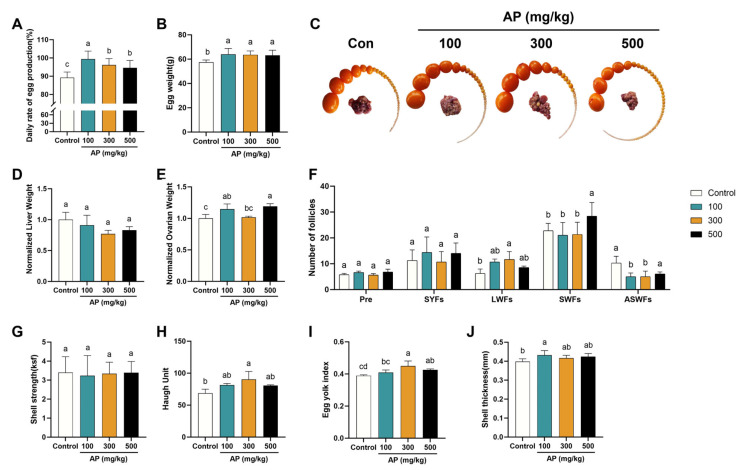
Effect of AP supplementation on the laying performance in D480 chickens. (**A**) Daily egg production rate of the laying chickens recorded for each group over a 21-day period. (**B**) Cumulative egg weight measured over the 21-day experimental period. (**C**,**F**) Numbers of follicles at different hierarchical grades in each group. Pre: Preovulatory follicles. SYFs: Small yellow follicles. LWFs: Large white follicles. SWFs: Small white follicles. ASWFs: Atretic small white follicles. (**D**) Liver weight normalized to the control group. (**E**) Ovary weight normalized to the control group. (**G**–**J**) Effects of AP supplementation on egg physical quality. Significant differences between groups are indicated by distinct lowercase letters (*p* < 0.05).

Compared with the control group, the daily egg production rate was significantly elevated by AP supplementation (Figure 9A), and egg weight was increased in all three AP-supplemented groups (Figure 9B).

Analysis of follicular distribution revealed that AP supplementation increased the number of LWFs and reduced the number of atretic small white follicles (ASWFs) relative to the control group (Figure 9C,F). No significant differences were observed in relative liver weight among groups (Figure 9D), whereas relative ovary weight was significantly elevated in chickens receiving 500 mg/kg AP (Figure 9E). Regarding egg quality traits, the Haugh unit was significantly improved only in the 300 mg/kg group, while the yolk index was elevated in both the 300 and 500 mg/kg groups. In contrast, eggshell thickness was significantly enhanced in the 100 mg/kg group, whereas no significant differences in eggshell strength were detected among treatments (Figure 9G–J).

## 4. Discussion

In the poultry industry, declining egg production during the late laying period represents a major economic challenge, largely attributable to ovarian aging. This process is characterized by a progressive loss of the follicular reserve, manifested by accelerated atresia of SWFs and aberrant apoptosis of GCs [1,5,25]. At the cellular level, this senescence is often triggered by the accumulation of oxidative damage and metabolic dysfunction, which compromise the energetic homeostasis and structural integrity of GCs [26]. Oxidative stress, characterized by an imbalance between ROS and antioxidant defenses, plays a central role in ovarian aging. Excessive ROS accumulation disrupts oxidative protein folding within the ER, leading to a loss of proteostasis [27]. This disturbance triggers ERS, which impairs cellular function and accelerates follicular atresia.

To investigate these mechanisms, the D-gal-induced aging model has been widely adopted in avian research. Chronic exposure to D-gal mimics natural aging by inducing systemic oxidative stress and the formation of advanced glycation end-products, thereby simulating the microenvironment of senescent ovaries in vitro [28,29]. In the present study, D-gal exposure successfully induced hallmark aging phenotypes in SWFs, including reduced proliferation and increased apoptosis. We therefore investigated AP, a natural bioactive flavonoid, as a potential intervention. AP is recognized for its robust antioxidant and cytoprotective capacities across various animal models [30,31]. Our in vitro results demonstrated that AP effectively alleviated D-gal-induced cytotoxicity, restored GC viability, and maintained follicular morphological integrity. These findings align with recent studies showing that other polyphenols, such as nobiletin and resveratrol, delay ovarian aging in laying chickens by mitigating oxidative stress [4,24], thereby supporting AP as a promising candidate for extending the reproductive lifespan of poultry.

To elucidate the molecular basis of AP-mediated protection, RNA-seq was performed to link the observed phenotypes with their underlying regulatory pathways. Differential expression analysis demonstrated that D-gal exposure profoundly disrupted cellular homeostasis, with significant enrichment of genes involved in the ERS pathway. The ER serves as the central organelle for protein folding and Ca^2+^ storage, and its functional decline is increasingly recognized as a critical driver of follicular atresia in aged laying chickens [7,32]. In high-producing laying chickens, the high metabolic demand for yolk protein synthesis places a substantial burden on the ER. As senescence advances, the decline in ER protein-processing capacity progressively activates the UPR cascade. Although the UPR initially functions as a compensatory mechanism to restore protein homeostasis, sustained stress ultimately drives a transition to pro-apoptotic ERS via the GRP78/CHOP signaling axis [33]. Consistent with this mechanism, our study demonstrated that AP treatment markedly attenuated the D-gal-induced transcriptional and translational upregulation of ERS-associated genes. Collectively, this evidence suggests that the cytoprotective effects of AP extend beyond general radical scavenging, specifically involving the restoration of ER functional stability and mitigation of stress-induced proteotoxicity. This mechanism is consistent with recent findings in melatonin-treated aged hens, in which alleviation of ERS was shown to be pivotal for rescuing atretic follicles [7].

Maintaining Ca^2+^ homeostasis is a prerequisite for cellular viability and is indispensable for the initiation and propagation of intracellular signaling networks that govern cell fate [34]. The disruption of Ca^2+^ homeostasis is increasingly recognized as a central mechanism linking oxidative stress to cellular aging [13,35]. Excessive Ca^2+^ release from the ER via IP_3_R channels can induce mitochondrial Ca^2+^ overload, amplify oxidative stress, and activate apoptotic signaling [36]. This study demonstrates that D-gal exposure triggers an aberrant surge in cytosolic Ca^2+^ levels in SWFs. This ionic disruption is intrinsically linked to overactivation of IP_3_R, the primary channel responsible for mobilizing Ca^2+^ from ER stores. Excessive efflux of Ca^2+^ via IP_3_R not only results in cytoplasmic Ca^2+^ overload, but also depletes ER luminal Ca^2+^ concentrations. Because the ER requires a high-Ca^2+^ environment for the proper protein folding by Ca^2+^-dependent chaperones, this depletion directly initiates ERS and the subsequent apoptotic cascade. AP treatment significantly downregulated *ITPR1* transcription and normalized the expression of other key Ca^2+^-handling genes (*CACNA1C*, *CACNA1D*, *CAMKII*, *MCU*, and *ATP2B1*). To further verify the mechanistic involvement of the IP_3_R-mediated signaling pathway, IP_3_/AM was employed. Activation of IP_3_R completely abolished the cytoprotective effects of AP, as evidenced by the reactivation of ER stress markers and apoptotic signaling, thereby confirming that the protective action of AP is dependent on IP_3_R-mediated Ca^2+^ regulation. These results findings are consistent with research in porcine models demonstrating that stabilization of ER Ca^2+^ flux is essential for maintaining follicular viability [37], and with prior reports showing that IP_3_R-mediated cytosolic Ca^2+^ overload accelerates cellular senescence [38]. Furthermore, the flavonoid astilbin has been reported to protect chicken embryonic cardiomyocytes by preserving Ca^2+^ homeostasis and attenuating ERS-induced injury [39], further supporting the conclusion that AP exerts its anti-apoptotic effects primarily by inhibiting IP_3_R-mediated Ca^2+^ release and subsequent ERS activation.

To bridge these mechanistic insights with practical poultry production, we conducted a 21-day feeding trial in naturally aged laying chickens. Dietary supplementation with AP significantly ameliorated age-associated declines in laying performance and ovarian function. Notably, AP administration markedly enhanced both the number and recruitment efficiency of SWFs, which constitute the primary pre-hierarchical follicular reserve in birds. Efficient recruitment of SWFs into the pre-ovulatory hierarchy is a key indicator of ovarian reserve status and is essential for sustaining long-term reproductive capacity [1,3].

The expansion of this follicular pool appears to result from AP-mediated suppression of apoptosis and preservation of GC layer structural integrity, as demonstrated by morphological and TUNEL analyses. Furthermore, AP supplementation significantly elevated serum P concentrations. P secretion is not only as a hallmark of GCs differentiation but also a functional prerequisite for the pre-ovulatory luteinizing hormone surge and subsequent ovulation [23]. The enhancement of P secretion therefore supports the notion that AP-mediated restoration of Ca^2+^ homeostasis and alleviation of ERS do not merely prevent cell death, but actively optimize the endocrine functionality of the senescent ovary. These observations are consistent with previous reports showing that dietary flavonoids improv egg quality and ovarian antioxidant capacity in late-laying chickens through modulation of cellular stress responses [6]. Collectively, these findings highlight AP as a safe and effective nutritional intervention capable of mitigating ovarian aging and sustaining reproductive performance in laying chickens, likely through modulation of ERS via IP_3_R-mediated Ca^2+^ signaling and maintenance of ovarian functional homeostasis.

## 5. Conclusions

**Figure 10:**
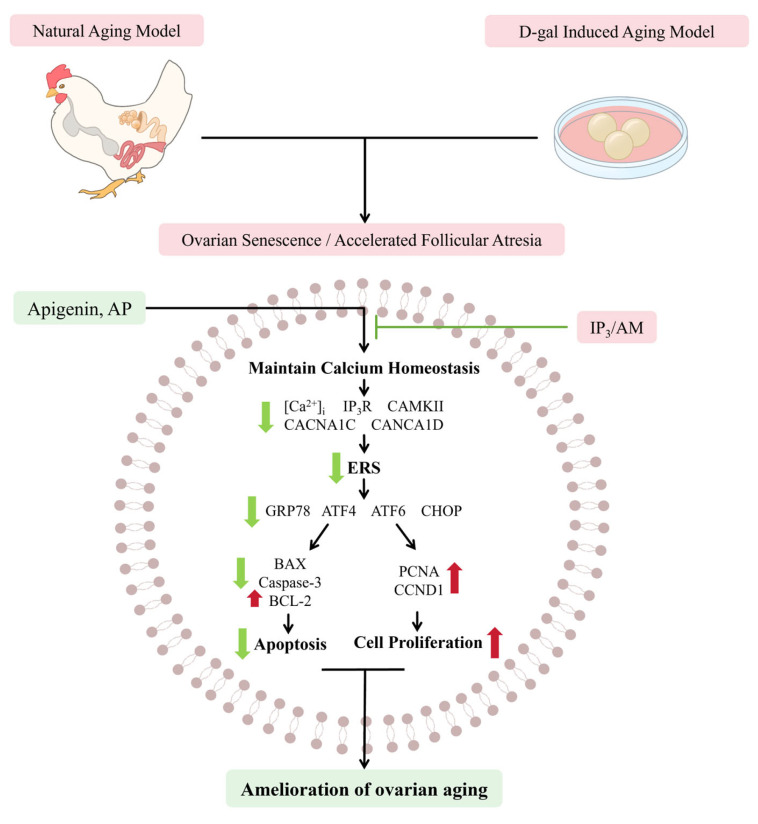
AP attenuates ovarian aging by regulating Ca^2+^-mediated endoplasmic reticulum stress in laying chickens.

This study demonstrates that the natural flavonoid AP alleviates ovarian aging by modulating IP_3_R-mediated Ca^2+^ signaling, thereby restoring intracellular Ca^2+^ homeostasis, attenuating ERS, and ultimately preventing follicular atresia. These findings indicate that AP represents a promising dietary supplement for improving ovarian health and sustaining reproductive performance. Future studies should focus on elucidating the crosstalk between IP_3_R and other Ca^2+^ regulators, as well as evaluating the bioavailability of AP (Figure 10).

## Figures and Tables

**Table 1:** Effects of AP supplementation on serum biochemical parameters.

Item	Control	100 mg/kg AP	300 mg/kg AP	500 mg/kg AP	*p*-Value
Ca^2+^ (mmol/L)	7.58 ± 0.127	8.26 ± 0.525	7.640 ± 0.143	7.350 ± 0.105	0.1829
ALT (U/L)	3.40 ± 0.240	4.00 ± 0.570	3.600 ± 0.510	2.500 ± 0.290	0.1543
AST (U/L)	209.00 ± 4.10	216.20 ± 5.020	224.8 ± 6.220	198.3 ± 12.540	0.1410
GGT (U/L)	25.40 ± 3.187	26.50 ± 3.379	22.00 ± 3.347	22.20 ± 2.922	0.7000
Alb (g/L)	18.78 ± 0.296	18.72 ± 0.470	18.60 ± 0.285	18.78 ± 0.601	0.9881
Glob (g/L)	40.70 ± 1.218	42.48 ± 1.188	41.02 ± 1.304	40.54 ± 0.546	0.5990
TP (g/L)	59.48 ± 1.448	61.20 ± 1.383	59.62 ± 1.374	58.62 ± 1.364	0.6267
P (nmol/L)	1.13 ± 0.133 ^b^	3.53 ± 0.524 ^ab^	5.20 ± 0.815 ^a^	5.30 ± 0.848 ^a^	0.0078

^a,b^ Means within a row with different superscripts differ significantly (*p* < 0.05).

## Data Availability

The original contributions presented in this study are included in the article/Supplementary Material. Further inquiries can be directed to the corresponding authors.

## Supplementary Materials

The following supporting information can be downloaded at: https://www.mdpi.com/article/10.3390/antiox15030323/s1, Table S1: Primer sequences for RT-qPCR.
