# Supplementary material for: Apigenin Prevents Ovarian Aging by Regulating Ca2+-Mediated Endoplasmic Reticulum Stress in Laying Chickens
Source: Antioxidants (Basel). 2026 Mar 4;15(3):323. doi: 10.3390/antiox15030323 (PMC13024370; doi:10.3390/antiox15030323)
Supplement: Supplementary file 1 [file antioxidants-15-00323-s001.zip › antioxidants-4170015-supplementary/Table S1. Primer sequences for RT-qPCR..pdf]

Table S1. Primer sequences for RT-qPCR.

Genes	Accession No.	Primer Sequence (5′-3′)	Product Size (bp)
PCNA	NM_204170.2	F: GGGCGTCAACCTAAACAGCA<br>R: AGCCAACGTATCCGCATTGT	97
CCND1	NM_205381.1	F: CCTCAAGAAAAGCCGGTTGC<br>R: CTGCGGTCAGAGGAATCGTT	86
CASP3	NM_204725.1	F: CAGCTGAAGGCTCCTGGTTT<br>R: GCCACTCTGCGATTTACACG	98
BCL2	NM_205339.2	F: ATCGTCGCCTTCTTCGAGTT<br>R: ATCCCATCCTCCGTTGTCCT	150
BAX	XM_040652724.2	F: CCCAGAGAGGGGAAACCAAC<br>R: CCACCTGGTCCTCTGCATTG	245
ITPR1	XM_040646075.2	F: AGCAAATGTACCAGGAGCCC<br>R: GGAGACCAGGGGAGGGTAAT	169
CACNA1C	NM_001396677.1	F: CAAGTCCTTCCAGGCTCTCC<br>R: GCTTCATACTGGTGCGCTTC	737
CACNA1D	XM_040646099.2	F: CCGAAGACAGCGCAAGAATC<br>R: TGGCCACTAACCTCTTGCAG	681
ATP2B1	XM_015276730.4	F: AGCCAGCACCATGATATACGA<br>R: ACACCCTTTTCCTGAAAATACAGC	901
CAMKII	XM_046943016.1	F: ACATCCATGGGTCTGCCAAC<br>R: CTGGAGTTAGCAAGGGCTCC	590
ATF4	NM_204880.3	F: TCACCCAATGACAACCCG<br>R: TCACCTTTGCTGACGCTACC	100
ATF6	XM_040677275.2	F: CGTCGTCTGAACCACTTACTGA<br>R: CCTTCTTTCCTAACAGCCACAC	101
GRP78	NM_205491.2	F: GATTGGACAAGAGAGAGGGTGA<br>R: CCATAACACGCTGGTCAAAGTC	162
CHOP	XM_015273173.1	F: TGGATGAGACACTGAATGC<br>R: CTTCCGCTTTGTCCTCTG	143
β-actin	NM_205518	F: ACACCCACACCCCTGTGATGAA<br>R: TGCTGCTGACACCTTCACCATT	136
